# The mast cell integrates the splanchnic and systemic inflammatory response in portal hypertension

**DOI:** 10.1186/1479-5876-5-44

**Published:** 2007-09-24

**Authors:** María-Angeles Aller, Jorge-Luis Arias, Jaime Arias

**Affiliations:** 1 Surgery I Department, School of Medicine, Complutense University of Madrid, Spain; 2 Psychobiology Department, School of Psychology, University of Oviedo, Asturias, Spain

## Abstract

Portal hypertension is a clinical syndrome that is difficult to study in an isolated manner since it is always associated with a greater or lesser degree of liver functional impairment. The aim of this review is to integrate the complications related to chronic liver disease by using both, the array of mast cell functions and mediators, since they possibly are involved in the pathophysiological mechanisms of these complications. The portal vein ligated rat is the experimental model most widely used to study this syndrome and it has been considered that a systemic inflammatory response is produced. This response is mediated among other inflammatory cells by mast cells and it evolves in three linked pathological functional systems. The nervous functional system presents ischemia-reperfusion and edema (oxidative stress) and would be responsible for hyperdynamic circulation; the immune functional system causes tissue infiltration by inflammatory cells, particularly mast cells and bacteria (enzymatic stress) and the endocrine functional system presents endothelial proliferation (antioxidative and antienzymatic stress) and angiogenesis. Mast cells could develop a key role in the expression of these three phenotypes because their mediators have the ability to produce all the aforementioned alterations, both at the splanchnic level (portal hypertensive enteropathy, mesenteric adenitis, liver steatosis) and the systemic level (portal hypertensive encephalopathy).

This hypothetical splanchnic and systemic inflammatory response would be aggravated during the progression of the chronic liver disease, since the antioxidant ability of the body decreases. Thus, a critical state is produced, in which the appearance of noxious factors would favor the development of a dedifferentiation process protagonized by the nervous functional system. This system rapidly induces an ischemia-reperfusion phenotype with hydration and salinization of the body (hepatorenal syndrome, ascites) which, in turn would reduce the metabolic needs of the body and facilitate its temporary survival.

## Background

Portal hypertension is a clinical syndrome defined by a pathological elevation in blood pressure in the portal system [1-3]. Ascites, portosystemic encephalopathy and variceal hemorrhage are some of its most notable clinical signs [4].

The increase in portal vein pressure is usually related to the obstruction of portal flow [5,6]. Depending on the level, the obstruction is classified as prehepatic, intrahepatic or posthepatic [7].

Prehepatic portal hypertension is most often caused by a cavernoma of the portal vein. This cavernoma is related to acute portal-vein thrombosis and develops concomitantly with splenomegaly, portosystemic shunts and the reverse flow in the unaffected intrahepatic portal veins [8]. It is considered that these patients have no underlying liver disease and their liver function is expected to remain normal throughout life [5,8].

Intrahepatic portal hypertension is most often caused by chronic liver disease, with the majority of preventable cases attributed to excessive alcohol consumption, viral hepatitis, or non-alcoholic fatty liver disease [9]. As a result, the pathology related to portal hypertension is associated with the pathology of chronic liver disease [10].

Post-hepatic portal hypertension, as the intrahepatic form is also associated with hepatocellular dysfunction [11].

Therefore, for the experimental study of portal hypertension, the prehepatic form is usually chosen since it has the least degree of hepatic affectation. In particular, the most frequently used experimental model of prehepatic portal hypertension is that which is achieved by partial portal vein ligation (PVL) in the rat [12-14].

The aim of this review is to integrate the complications related to chronic liver disease by using both, the array of mast cell functions and mediators, since they possibly are involved in the pathophysiological mechanisms of these complications.

## Experimental prehepatic portal hypertension

PVL in various animals, but particularly in the rat, has been widely used to study portal hypertension [12,14,15]. The surgical technique is simple. In brief, the rat is anesthetized and after laparotomy, the portal vein is dissected and isolated. A 20-gauge blunt-tipped needle is placed alongside the portal vein and a ligature is tied around the needle and the vein. The needle is immediately removed, yielding a calibrated stenosis of the portal vein [12].

**Figure 1:**
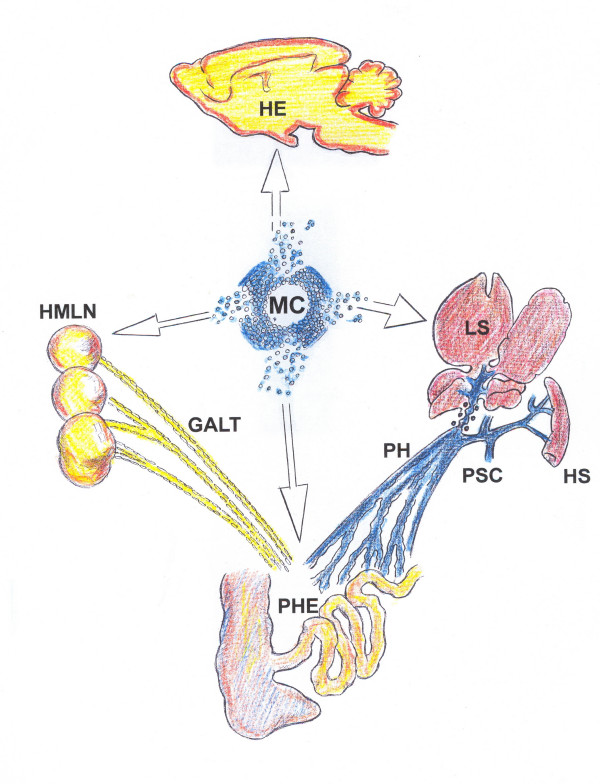
**Alterations related to prehepatic portal hypertension in the rat, where mast cells and their mediators could participate in its production**. The systemic inflammatory response would integrate splanchnic alterations (portal hypertensive enteropathy, hepatic steatosis, splenomegaly and portosystemic collateral circulation) with extrasplanchnic alterations (portal hypertensive encephalopathy).

If it is taken into account that the intensity of the portal hypertension is determined by the resistance to the inflow produced by the constriction of the portal vein, this model of prehepatic portal hypertension could be improved by increasing the initial resistance to the blood flow. With this objective in mind, we have modified the surgical technique by increasing the length of the stenosed portal tract with three equidistant partial ligations. In brief, three partial ligations are performed in the superior, medial and inferior portion of the portal vein, respectively, and maintained in position by their previous fixation to a sylastic guide. The stenoses are calibrated by a simultaneous ligation around the portal vein and a 20-G needle [16,17] (Figure 1).

At two weeks of evolution, portal hypertension is a consequence of a pathological increase in the portal venous inflow ("forward" hypothesis) and resistance ("backward" hypothesis) [18,19]. The increase in blood flow in the portal venous system is established through the splanchnic arteriolar vasodilation that produces hyperdynamic splanchnic circulation or splanchnic hyperemia [20]. In turn, the increase in vascular resistance to the portal blood flow is found in the presinusoidal (PVL) hepatic circulation, as well as the portal collateral circulation (enhanced portosystemic collateral resistance) [21,22]. Thus, normalization of elevated portal pressure can only be achieved by attempting to correct both, elevated portal blood flow and elevated portal resistance [21] (Figure 1).

The hyperdynamic circulation stands out among the systemic alterations related to portal hypertension [22]. This vasodilatory state in short-term (2–4 weeks) PVL rats has been principally attributed to two mechanisms: increased circulating vasodilators and decreased response to vasoconstrictors [22-24]. The vasodilators involved include nitric oxide (NO), carbon monoxide (CO), alpha-tumoral necrosis factor (TNF-α), glucagon, prostacyclin (PGI_2_), endothelium-derived hyperpolarizing factor, endocannabinoids, adrenomedullin and hydrogen sulfide (H_2_S) [22]. In turn, the hyporeactivity to the vasoconstrictors, that is, to endogenous ones (norepinephrine, endothelin, vasopressin) or exogenous (alpha agonists) reflect the impaired vasoconstrictor response, which contributes to vasodilation [25]. Furthermore, it is conceivable that there might be different mechanisms underlying the hyporeactivity of vasoconstrictors in portal hypertension.

**Figure 2:**
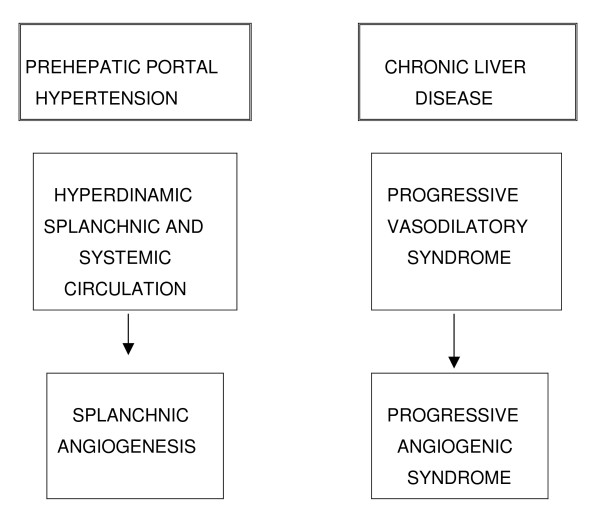
**Evolution of prehepatic portal hypertension**. Portal hypertension worsens when associates with chronic liver disease.

It has been proposed that the splanchnic and systemic vasodilation is the initial step leading to the hyperdynamic syndrome or progressive vasodilatory syndrome [22]. Multi-organ failure in chronic liver disease is in large part attributable to this syndrome [22,26] (Figure 2).

In the early evolutive phase of prehepatic portal hypertension in the rat, mainly two types of portosystemic collateral circulation are established: splenorenal and paraesophageal [27]. The development of the portal collateral venous system is not only due to the opening of preexisting vessels, but also to new vessel formation, which is a very active process. Particularly, it has been shown that portal hypertension in the rat is associated with VEGF (vascular endothelial growth factor) induced angiogenesis [28] (Figure 1).

An increased systemic angiogenic response is a common finding in patients with overt liver diseases or advanced cirrhotic stages [22,29-31]. In this late stage the impaired modulation of vascular growth with deregulation of vascular remodeling is a pathophysiological mechanism that not only participates in the production of splanchnic alterations (portosystemic collateral circulation, cirrhotic liver and hypertensive portal intestinal vasculopathy) [9,30,32] but also in different systemic alterations (hepatic encephalopathy, portopulmonary hypertension, vascular spiders and digital clubbing) [22,29,31,33].

Therefore, the angiogenic response developed in portal hypertension and mainly located in the splanchnic area seems to progress when it is associated with a chronic liver disease. In this way, the existence of a progressive angiogenic syndrome would be proposed that contributes significantly to structural splanchnic and systemic remodeling [34] (Figure 2).

The crosstalk between the vasodilator and angiogenic responses, both in prehepatic portal hypertension and in chronic liver failure, would be represented by the inflammatory response, established in both conditions. This relation would be based on the fact that both vasodilatory and angiogenic responses are also components of the inflammatory response [35-38].

## The inflammatory response

The successive pathophysiological mechanisms that develop in the interstitium of tissues when they undergo inflammation are considered increasingly complex trophic functional systems for using oxygen [36,38].

The nervous or immediate functional system presents ischemia-revascularization and edema, which favor nutrition by diffusion through the injured tissue. This trophic mechanism has a low energy requirement that does not require oxygen (ischemia) or in which the oxygen is not correctly used, with the subsequent development of reactive oxygen and nitrogen species (ROS/RNS) (reperfusion). In this phase, while the progression of the interstitial edema increases the space between the epithelial cells and the capillaries, the lymphatic circulation is simultaneously activated (circulatory switch). Thus, the injured tissues adopt an ischemic phenotype (hypoxia) [38].

In the following immune or intermediate phase of the inflammatory response, the tissues and organs which have suffered ischemia-reperfusion, are infiltrated by inflammatory cells and bacteria. Interstitial infiltration is favored by the actions of intrinsic and extrinsic components of the coagulation cascades. In the tissues and organs which suffer oxidative stress, symbiosis of the inflammatory cells and bacteria for extracellular digestion by enzyme release (fermentation) and by intracellular digestion (phagocytosis) could be associated with a hypothetical trophic capacity. Improper use of oxygen persists in this immune phase and is also associated with enzymatic stress. Furthermore, lymphatic circulation plays a major role and macrophages and dendritic cells migrate to lymph nodes where they activate T lymphocytes.

It is considered that angiogenesis characterizes the last or endocrine phase of the inflammatory response, so nutrition mediated by the blood capillaries is established.

**Figure 3:**
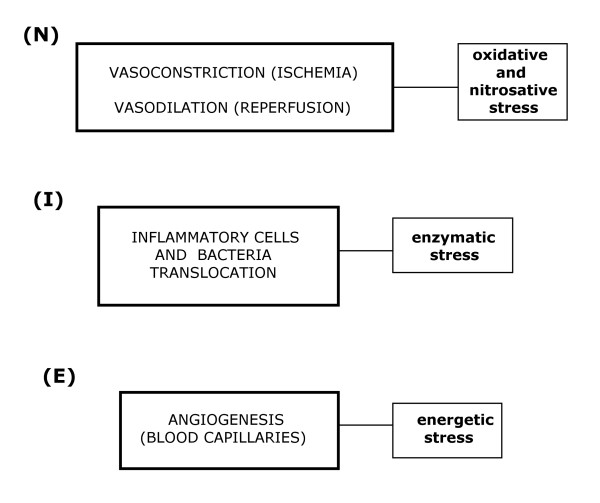
**Phases of the inflammatory response**. N: immediate or nervous; I: intermediate or immune. E: late or endocrine.

However, the angiogenic process becomes active early on and excessive proliferation of endothelial cells occurs which, in turn, develops a great density of endothelial sprouts. Through this initial and excessive proliferation, the endothelial cells could successively perform antioxidant and antienzymatic functions. These functions would favor the evolution of the inflammatory response towards tissue repair through specialized capillary development. If so, it would be in this last phase of the inflammatory response when the process of angiogenesis would be responsible for tissue nutrition through the capillaries. Oxygen and oxidative metabolism are an excellent combination through which the cells can obtain an abundant energy supply (energetic stress) for tissue repair by epithelial regeneration or wound healing [34,36-39] (Figure 3).

## The inflammatory response mediated by mast cells in experimental portal hypertension

PVL rats are far from having a uniform evolution, since they can present wide variability in both hepatic weight, or degree of liver atrophy [27], as well as in the type and degree of portosystemic collateral circulation developed [19,27]. Furthermore, the variability of this experimental model of prehepatic portal hypertension is not only observed in short-term evolution (14 to 28 days) which is where it is studied most, but also in the chronic evolutive stages (6 to 14 months) [40].

One of the reasons that the prehepatic portal hypertension experimental model presents great evolutive variability could be based on its inflammatory nature [34]. Thus, the pathogenic mechanisms proposed for the post-traumatic inflammatory response as unifiers of the phylogeny, and therefore with the category of generics [39], could also participate in the production of the alterations related to portal hypertension.

It has been considered that portal hypertension is essentially a type of vascular pathology resulting from the chronic action of mechanical energy on splanchnic venous circulation [41]. This kind of energy can stimulate the endothelium which, owing to its strategic position, plays an exceedingly important role in regulating the vascular system by integrating diverse mechanical and biochemical signals and by responding to them through the release of vasoactive substances, cytokines, growth factors and hormones [42-44]. Mechanical energy may also act in the vascular endothelium as stress stimuli, generating an inflammatory response [43-45]. If it is considered that, in the case of portal hypertension, there is an endothelial inflammatory response induced by mechanical energy that affects the splanchnic venous circulation and, by extension, the organs into which its blood drains, it could be speculated that there is a common etiopathogeny that integrates the pathophysiological alterations presented by these organs [41].

**Figure 4:**
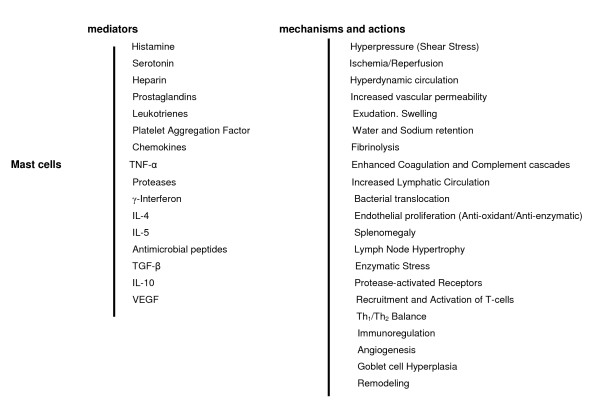
**Mast cell phenotypes**. Functional "plasticity" of mast cell during the splanchnic inflammatory response in portal vein ligated-rats.

Mast cells, strategically located close to blood vessels [46], could be among the first responders to the mechanical stimuli that initiate splanchnic inflammation in rats with prehepatic portal hypertension. When appropriately activated, mast cells have the ability to produce vasoactive amines, enzymes (proteases), cytokines, chemokines and growth factors through degranulation [46,47]. This plasticity of the mast cells, which is the base for the called "mast cell heterogeneity" [48] suggests that mast cells can also show diverse responsiveness during the splanchnic inflammatory response related to the pathological increase of portal pressure. Thus, during the inflammatory response evolution, genetic and environmental factors can position or "tune" mast cells within a broad spectrum of functional responsiveness [46]. If so, mast cells could successively participate, by piecemeal degranulation [46,47], in the expression of the three trophic functional systems, which have been previously proposed, as components of the inflammatory response [36,38] (Figure 4).

Mature mast cells can be found in almost every tissue, but they are preferentially localized in organs that are in contact with the environment, namely the skin, airways and gut [49]. That is why, when pathological splanchnic venous hypertension is produced, the gastrointestinal tract becomes an organ predisposed to host the inflammatory response mediated by its resident mast cells.

## Mast cells in portal hypertensive enteropathy

Mature mast cells, normally resident close to the gastrointestinal blood vessels and epithelia [46,49], immediately and directly suffer the sudden increase in venous pressure produced by the PVL in the rat.

In an early period, portal venous hyperpressure is highest [18,19] since portosystemic collateral circulation has not yet developed, and the intestinal mucosa ischemia is an immediate consequence of the venous stasis. Mucosal hypoxia is also related to the constriction of mucosal arterioles, meanwhile the dilation of arterioles in the muscularis increases the blood flow in this layer [50]. Hypoxia in the intestinal mucosa causes oxidative and nitrosative stress. However, though hypoxia inducible factor 1 (HIF-1), it also enhances the expression of hypoxia responsive genes, and therefore improves cell survival in conditions of limited oxygen availability [45].

Two days after PVL in the rat, portal hyperpressure is associated with intraperitoneal free exudates, peripancreatic edema, hypoproteinemia and hypoalbuminemia. The inflammatory nature of these alterations can be hypothesized, since the oral administration of budesonide prevents these early exudative changes [51]. The venous hyperpressure associated with hypoxia could be an important trigger of the splanchnic mast cell activation in the earlier periods of prehepatic portal hypertension in the rat. Degranulation of mast cells results in the release of preformed mediators such as histamine, a potent vasodilator and exudative mediator [49,52]. Histamine can cause exudation related to an endothelial permeability increase, which is the cause of swelling and production of peritoneal exudate in this early evolutive phase of experimental portal hypertension [51].

The inhibition of this acute inflammatory response by budesonide would indicate the efficacy of this steroid in the prophylaxis of this early acute response. It could be speculated that budesonide produces a down-regulation of the pro-inflammatory mediators partially due at least to an inhibitory effect on the transcription factors that regulate inflammatory gene induction, including activator protein-1 (AP-1) and NF-κB, and through mechanisms similar to those that also act with great efficiency on the allergic inflammatory response to allergens [53,54]. It has been also described that corticosterone has a rapid inhibitory effect on histamine release from rat peritoneal mast cells which the classical genomic mechanisms could not explain [55]. Taken all together, these results suggest that mast cells could be key cells in the early stages of acute portal hypertensive splanchnic inflammation (Figure 4).

We have shown that prophylaxis with Ketotifen, an anti-inflammatory drug that stabilizes mast cells [56], reduces portal pressure, the number of degranulated mast cells in the cecum and the concentration of mast cell protease II (RMCP-II) in the mesenteric lymphatic nodes of rats with early (48 hours) prehepatic portal hypertension [57]. Although histamine and serotonin stand out among the mediators released by mast cells and cause vasodilation and edema due to increased vascular permeability [46,48], neutral proteases may also regulate the tone of the splanchnic vascular bed and provoke matrix degradation and edema [58]. Particularly RMCP-II, considered a specific marker of rat mucosal mast cell degranulation, can modulate the vascular function through their ability to convert Angiotensin I to Angiotensin II. It also may promote epithelial permeability. Angiotensin II is a powerful vasoconstrictor that produces mucosal ischemia and also increases vascular permeability and promotes recruitment of inflammatory cells into tissues [59]. Furthermore, both Angiotensin II, which produces vasoconstriction and mucosal ischemia, and RMCP-II, which increases intestinal permeability and enhances antigen and bacteria uptake, consequently induce bacterial translocation to the mesenteric lymph nodes where they would activate a "chemotactic call" to mast cells and worsen the inflammatory responses [60]. Therefore, Ketotifen could inhibit mast cell migration and activation in the mesenteric lymph nodes and thus reduce the release of mediators involved in the development of the increased portal venous inflow that causes portal hypertension in short-term PVL rats [57].

In a later evolutive phase (4 weeks) portal hypertension is associated with features of hyperdynamic circulation [18,19]. In this model of pre-hepatic portal hypertension, splanchnic and systemic vasodilation is the initial step leading to the hyperdynamic syndrome [22]. Also the hyperdynamic circulation could favor the maintenance of the inflammatory response that has been proposed as characteristic of this experimental model. [34,41]. Particularly, the hyperdynamic splanchnic circulation could be involved in the persistence of a low grade gastrointestinal inflammation. First, since the pathological increase of the portal pressure associated to the hyperdynamic splanchnic circulation, could favor a disturbed splanchnic venous flow, with shear stress mediated by non-laminar flow [22]. Unidirectional laminar shear stimulates production of NO and in the long-term decreases ROS production and has anti-inflammatory effects [61,62]. In contrast, non-laminar flow or disturbed flow associated with low shear stress have profound effects on biology of the vascular wall, particularly the vascular endothelium, and could stimulate inflammation [61]. Second, both the increase in blood flow speed and the opening of the arterio-venous shunts that induce the splanchnic hyperdynamic circulation, would reduce the oxygen tissue availability. This fact would induce tissue hypoxia and, therefore the chronicity of the inflammatory response (Figure 4).

Splanchnic and systemic hyperdynamic circulation, related to progressive vasodilation and the development of arteriovenous splanchnic and systemic shunts, are associated with water and sodium retention [14,22,26]. The early development of portosystemic collaterals in PVL rats associated with the splanchnic hyperdynamic circulation could fundamentally represent the switch of the splanchnic portal system in a big arterio (splanchnic)-venous (systemic) shunt. Thus, progressive hyperhydration and salinization of the body produces the expansion of the plasma volume and play a fundamental role in perpetuating and aggravating the hyperdynamic syndrome [22,26] (Figure 4).

Portal hypertensive rats at six weeks of evolution show increased mast cell infiltration in the duodenum, jejunum, ileum and superior mesenteric lymph node complex [63,64]. Mast cells are normally found in great density in the mucosa of the gastrointestinal tract [65]. This accumulation at tissue sites where foreign materials attempt to invade the host suggests that mast cells are among the first cells to initiate defense mechanisms [66-68]. This function of mast cells in the gastrointestinal tract, which provides a barrier against infection, could explain their increase in the small bowel in rats with prehepatic portal hypertension [64].

Mast cells have the unique capacity to store presynthesized TNF-α and thus can spontaneously release this cytokine after they are activated [69]. Therefore, the excess number of mast cells in the small bowel and in the mesenteric lymph node complex of PVL rats could be related to their ability to release the stored TNF-α when an appropriate stimulus is acting. It has been hypothesized that TNF-α causes vasodilation through both the PGI_2 _and NO pathways [69]. If so, the release of the stored TNF-α by activated mast cells may be involved in the development of the hyperdynamic splanchnic state [70].

Maybe mast cells also contribute to avoid the risk of hemostasis and portal thrombosis in PVL rats, which would be related to the splanchnic hemodynamic impairments, through the expression of tissue type plasminogen activator (t-PA) which induces fibrinolysis and heparin production [71]. The convergence between the clotting system and complement [72], both of which form proteolytic cascades, probably originated from a common ancestral developmental-immune cascade [73], which suggests that their activation occurs simultaneously in this experimental model of prehepatic portal hypertension. This concurrent activation of hemostasis and Complement cascades, could be regarded as a critical mediator of mast cell activation [65,68,72,74].

Portal hypertension is one factor determining bacterial intestinal translocation [74]. A reduced bacterial translocation rate is recorded in simple PVL rats [75]. However, in triple-PVL- rats it has been shown that the incidence of intestinal bacterial translocation to mesenteric lymph nodes increases significantly [76]. Mast cells can prevent dangerous bacterial translocation in PVL rats developing thus a protective role in defense against infections [46,67,68]. In this way, mast cell activation could be regarded in PVL rats in the context of antibacterial host defense preserving the rat life during the course of innate and adaptive host response against pathogens [68] including modulation of both dendritic cell and T-cell responses [77-79]. Growing evidence suggests physiological roles for intestinal mast cells in the protection of tissues from inflammatory damage [65,67,68,77,79].

In addition to initiating innate immune response in the gastrointestinal tract [46,52,60], mast cells are central in the initiation and regulation of the adaptive immunity [52,68,69,79,80]. Mast cells by TNF-dependent [81,82] and TNF-independent (complement) [83] mechanisms induce hypertrophy of the draining lymph nodes. Both mechanisms could be involved in the mesenteric lymph node hypertrophy that the PVL rats present (Figure 1) since in the absence of mast cells, bacteria and their products alone do not initiate nodal hypertrophy [81].

The increased presence of mast cells in the hypertrophied mesenteric lymph nodes of PVL-rats has been suggested, which could be related to migration from the inflamed intestine. In turn, the activation of the mast cells in the mesenteric lymph nodes in these rats with portal hypertension, would not only collaborate in the production of mesenteric adenitis, but also would constitute a source of inflammatory mediators located between the intestine and systemic blood circulation [64].

The lymph tissue associated with the intestine constitutes the largest lymphatic organ of the body and its activation in portal hypertensive enteropathy would produce the release of inflammatory mediators [84]. These would be transported by the intestinal lymph vessels to the pulmonary circulation-inducing and inflammatory phenotype- and later to the systemic circulation. The priority of mesenteric lymphatic circulation with respect to portal circulation for transporting pro-inflammatory mediators released in the intestinal wall in different conditions related to intestinal ischemia, such as hemorrhagic shock or serious burns [85], suggests that in other conditions that also produce a hyperdynamic splanchnic state with intestinal ischemia, like prehepatic portal hypertension, the mesenteric lymph is a regional pro-inflammatory mediator vehicle, that is, a splanchnic one, but with a systemic effect [41] (Figure 4).

Due to the destructive potential of proteases, they have been considered to act primarily as degradative enzymes in the interstitial space [86,87]. However, these enzymes make important contributions to intestinal immune response [52,88] and that is why they could collaborate in the production of portal hypertension enteropathy mediated by mast cells. Tissue responses to these enzymes are modulated by protease-activated receptors (PARs) [89], a new subfamily of G protein-coupled receptors that use a fascinating mechanism to convert an extracellular proteolytic cleavage event into a trans-membrane signal [90]. RMCP-II, a product of rodent mast cell degranulation, in addition to other serine proteases such as thrombin and tripsin, through PARs activation, may modulate the splanchnic immune response in PVL rats. The demonstration that PARs may link mast-cell derived proteases to experimental bladder inflammation [90] is the basis for this hypothetical relation.

After activation in the intestine of PVL rats, mast cell could migrate, via afferent lymphatics to mesenteric lymph nodes where they mediate T lymphocyte recruitment to facilitate antigen presentation and the initiation of an adaptive response. The contribution of mast cells to the induction of this immune response has been demonstrated during the sensitization phase of dinitrofluorobenzene-induced contact hypersensitivity in mice [91]. In this study it is not only demonstrated that fluorescent-labeled mast cells injected in the skin appeared in draining lymph nodes after antigen application, but also that they subsequently migrate to the spleen [91].

Spleen enlargement is often detected in PVL rats accompanied by the rise in portal venous pressure [40,41]. However, congestion cannot be considered as the only cause of splenomegaly, since other pathogenic mechanisms, including the immune ones, participate in its production [92,93]. Rats with bile duct ligation present hypodynamic intrasplenic circulation, associated with decreased TNF-α and eNOS phosphorylation and increased VEGF expression [94].

Histamine is synthesized and stored in the vesicles of mast cells [46,49,79] and is involved in regulation and modulation of immune response through the stimulation of four subtypes of receptors present on the target cells [95]. It has been speculated that higher content of histamine in the spleen could attenuate the immunological response through histamine receptors in T lymphocytes, macrophages and mast cells. Moreover, higher content of histamine in spleen may possibly change the Th1/Th2 balance through histamine receptors in T lymphocytes [95]. In portal hypertensive-rats at six weeks of evolution, the increase in diameter and number of blood vessels in the submucosa has already been shown in the duodenum, which at the same time is correlated with mast cell infiltration [63]. Therefore, vasodilation and angiogenesis, which are responsible for the increase in size and number of vessels, and in turn, for vascular structural alterations that characterize portal hypertensive enteropathy [96-98] can be attributed to, among other factors, the pathophysiological effects produced by the excessive release of mast cell mediators [63,64] (Figure 4).

Since 1985 when McCormack et al. [99] described hypertensive gastropathy in patients with portal hypertension, successive histological studies on the remaining portions of the gastrointestinal tract have demonstrated that alterations similar to gastric ones are found in the duodenum, jejunum, ileum, colon and rectum [97,98]. Since the basic structural alteration found in the gastrointestinal tract is vascular and consists of increased size and number of the vessels, the very appropriate name of "hypertensive portal intestinal vasculopathy" has been proposed [96].

The ability of the mast cells for the synthesis and selective or dedifferentiated release of different mediator molecules of the inflammatory response [46,79] would explain their participation in the different evolutive phases of the portal hypertensive enteropathy. In particular, in the last phases, the chemotactic factors derived from the mast cells stimulate the proliferation of fibroblasts and the synthesis of collagen. Meanwhile, histamine and heparine promote the formation of new blood vessels. Both fibrogenesis and angiogenesis are responsible for fibromuscular and vascular proliferation in the intestinal wall, respectively [41].

Splanchnic hyperemia, increased splanchnic vascularization and the development of portal-systemic collateral circulation in portal hypertensive rats are partly a VEGF-dependent angiogenic processes [28,100]. Extrahepatic portosystemic collateral circulation persists in long-term (3, 6 and 12 months) PVL rats [17,27]. However, in these chronic evolutive phases, although the animals present collateral circulation, this is not always associated with portal hypertension [40,41]. That is why it has been proposed that long-term collateral vasculopathy in PVL rats constitutes a remodeling process not associated with portal hypertension [101].

Ischemic mucosal injury could be the main inducing stimulus for the expression of the potent angiogenic factor VEGF in the intestine of PVL rats and, therefore one of the most important mediators in the production of hypertensive portal intestinal vasculopathy in this experimental model [63,64]. And so mast-cell derived histamine has a variety of functions in the inflammatory response regulation including VEGF production via H_2 _receptor stimulation [102]. Also production and release of angiogenic (VEGF) and growth factors by mast cells [46] in the intestinal mucosa and submucosa of PVL rats could have a role in modulating this splanchnic angiogenic process [41,103].

The angiogenic hyperactivity that occurs in the prehepatic portal hypertensive model, could constitute an extending and progressively intense process or a "progressive angiogenic syndrome". That is, it would initiate in the splanchnic area (portosystemic collateral circulation, splenomegaly and intestinal vasculopathy) and it would reach a systemic diffusion during its evolution (peripheral systemic vasculopathy affecting the extrasplanchnic organs).

The earliness, intensity and diffusion which establishes angiogenic hyperactivity in PVL rats, suggests that the endothelial cells could carry out other functions aside from forming new blood vessels. And so, the anti-oxidant and anti-enzymatic properties of the endothelium could stand out [104]. The expression of anti-oxidant and anti-enzymatic functions by the endothelial cells during the evolution of the splanchnic inflammatory response in PVL rats would therefore represent a defensive mechanism. This early hypothetical endothelial proliferation would constitute a local mechanism to counteract both oxidative stress and enzymatic stress, inherent to the inflammatory response (Figure 4).

The angiogenic response also contributes significantly to structural splanchnic and systemic remodeling. The structural changes that are produced in the long-term in prehepatic portal hypertension in the rat, could be similar to those described in other chronic inflammatory processes. These morphological alterations would not only be vascular, both macro and microscopic, but also the rest of the intestinal structures would participate in greater or lesser intensity [105]. In particular, the morphological vascular alterations stand out in chronic portal hypertensive enteropathy [96-98]. However, we have also described, in the experimental chronic portal hypertensive enteropathy, the existence of epithelial remodeling, which consists in goblet cell hyperplasia [105,106]. Goblet cell hyperplasia with mucus hypersecretion is an alteration characteristic of epithelial remodeling of the respiratory tract in chronic inflammatory processes, as are asthma and chronic obstructive pulmonary disease [107,108]. And so, goblet cell hyperplasia could be attributed to chronic hypertensive portal enteropathy in the rat [105,106]. Mucus secreted by goblet cells into the intestinal lumen, constitute a component of the mucosal defense [67]. But also goblet cells can secrete into the lumen of the small and large intestine trefoil peptides. These peptides are protease-resistant factors and their presence both protects against intestinal epithelial injury and promotes repair [109].

Therefore, mast cells and their mediators could participate in the production of morphological alterations characteristic of the splanchnic remodeling associated with experimental prehepatic portal hypertension [41].

And so it could be considered that in prehepatic portal hypertension in the rat, a low-degree chronic splanchnic inflammatory response is produced that would evolve in three dominating successive expression of pathological functional systems called the nervous, immune and endocrine. Since mast cells are functionally heterogeneous and participate in the establishment of these three systems, it could be considered that their intervention in this type of splanchnic inflammatory response is indispensable.

## Mast cells in portal hypertensive encephalopathy

Prehepatic portal hypertension in humans is associated with neuropsychological and brain magnetic resonance changes consistent with minimal hepatic encephalopathy [110]. Since intrinsic hepatocellular disease does not exist in this type of portal hypertension, the existence of a portal-systemic bypass is the principal cause of minimal hepatic encephalopathy. Consequently, this encephalopathy is categorized as type B [111].

The PVL rat model could be appropriate for the experimental study of the minimal hepatic encephalopathy related to prehepatic portal hypertension because portal-systemic shunting is developed [12,18]. Hence, it should be considered that an associated hepatic pathology exists [41] (FIGURE 1).

A histological study of the liver of PVL rat, has demonstrated that hepatocytic fatty infiltration exists. Fat accumulation in the hepatocytes progress from a short- (1 month) to a long-term (1 year) evolutive stage of portal hypertension and thus the persistence of etiopathogenic mechanisms involved in its production could be considered. Therefore, it could also be considered that PVL in the rat not only makes it possible to obtain an experimental model of portal hypertension but also a steatosis model [41,112-114].

The important role that inflammation has on modulatingthe molecular pathogenesis of hepatic encephalopathy has recently been highlighted [33]. Inflammation, however, may not only be limited to modulating the severity of hepatic encephalopathy but also could indeed be its own pathophysiological mechanism [33,34]. If so, the inflammation of the central nervous system, when related to prehepatic portal hypertension [34], could be the basic mechanism that drives the essential nature of minimal hepatic encephalopathy [34].

At one month of evolution, prehepatic portal hypertensive-rats present increased SDF-1 alpha levels in the hippocampus and cerebellum associated with increased TNF-α and CXCR4 levels in the hippocampus [115]. The increase of the chemokine system CXCR4/SDF-1 alpha in the hippocampus could be related to a remodeling structural process since SDF-1 alpha, a pro-inflammatory cytokine, regulates neurodevelopmental processes in the central nervous system and neuronal migration [116].

Chemokines have a dual role as neurodegenerative or neuroprotective molecules in the central nervous system. In experimental portal hypertensive encephalopathy, chemokines can contribute to creating an immune phase in the hippocampus and cerebellum that does not necessarily involve only harmful phenomena, but rather exerts a beneficial remodeling action [34]. The objective would be to adapt cerebral areas to the new metabolic state created by portal hypertension [33]. At the same time, the brain changes demonstrated in this experimental model of portal hypertension could be related to the development of a minimal hepatic encephalopathy [115].

Mast cells can be found in areas of the central nervous system of many mammalian species. Mast cells in rats are predominantly located in the thalamic region of the brain [117,118]. Since these cells, when activated, could translocate from the splanchnic area to the central nervous system [119] we have hypothesized that mast cells would be involved in a splanchnic-brain chemokine-mediated crosstalk [115]. Mast cells can migrate from the splanchnic region to the brain, and release different neurotransmitters and neuromodulators such as monoamines, proteases, cytokines and histamine [118]. Mast cell-derived products also can enter neurons by a process termed transgranulation, a novel form of brain-immune system communication [120].

Since the gastrointestinal tract is known to contain the most extensive immune system in the body as well as the largest and most diverse collection of nerves outside the central nervous system, there is an ample opportunity for these inflammatory cells to interact with neurons [121]. In this way, the enteric nervous system is considered a local "minibrain" [122]. Hence, mast cell degranulation can release mediators that can signal intrinsic and extrinsic neurons [122] and also provide a connection node between the central nervous system and enteric nervous system [121,122]. The evidence for a bidirectional crosstalk between mucosal mast cells and the enteric and central nervous systems [121,122] suggest that these inflammatory cells are potential integrators of the systemic inflammatory response that induces portal hyertension.

## Chronic liver disease and portal hypertension

The functional ability of the liver could be considered one of the most important factors for modulating the evolution of the syndrome induced by portal hypertension. Particularly, chronic liver disease and cirrhosis can aggravate the portal hypertensive inflammatory syndrome exceedingly [34].

The most studied models of cirrhosis in the rat are those achieved by extrahepatic cholestasis [14,123,124], and by administering carbon tetrachloride (CCl_4_) [14,125] or (TAA) [101,126]. Hepatic fibrogenesis is the common final result of injury to the liver. Furthermore, fibrosis is believed to be a critical factor that leads to hepatic dysfunction [127].

Hepatic dysfunction related to fibrosis or cirrhosis in the rat would aggravate the grade of systemic inflammation characteristic of prehepatic portal hypertension and as a result would increase the incidence of complications [34]. It has been recognized that liver mast cells are present under normal and pathological conditions in both humans and experimental animals [128]. There is evidence that mast cells are involved in various hepatobiliary disorders [128-130]. Mast cells have been shown to promote fibroblast proliferation. They are found in the periportal sinusoids joining the destruction and inflamed limiting plates in chronic liver diseases and biliary/cholestatic diseases, suggesting that they are at least involved in inflammation and periportal fibrosis [128,131,132]. Therefore, mast cells can be considered key elements during liver fibrosis of any etiology [132,133].

Mast cells also are involved in the regulation of physiological and pathological hepatic vasculogenesis to which they contribute by producing mediators such as heparin, histamine, tryptase, TNF-α, transforming growth factor-beta (TGF-β) and VEGF [46,49]. In this way, it has been hypothesized that mast cells may be primary elements in the transition from sinusoidal to capillary-type endothelial cells [134]. Mast cell mediated diffuse hepatic sinusoidal capillarization may therefore be pathogenically significant in the progression of liver disease [134].

One important consequence of chronic liver disease is that both the local and systemic anti-oxidant function of the liver is reduced [135]. A decreased anti-oxidant capacity of the liver plays an important role in the pathogenesis of liver fibrosis or cirrhosis and portal hypertension [136,137]. That is why, anti-oxidants have been proposed as an adjunctive therapy in the treatment of portal hypertension [135,136]. However, the deficient anti-oxidant ability of the liver when suffering from fibrosis or cirrhosis could also induce the production of a systemic pathology. In this hypothetical situation, in prehepatic portal hypertensive-rats with chronic oxidative stress and a low-grade inflammatory state, the reduction of the hepatic anti-oxidant capacity would increase the intensity of the inflammatory systemic response and add severity to this syndrome [34,40].

**Figure 5:**
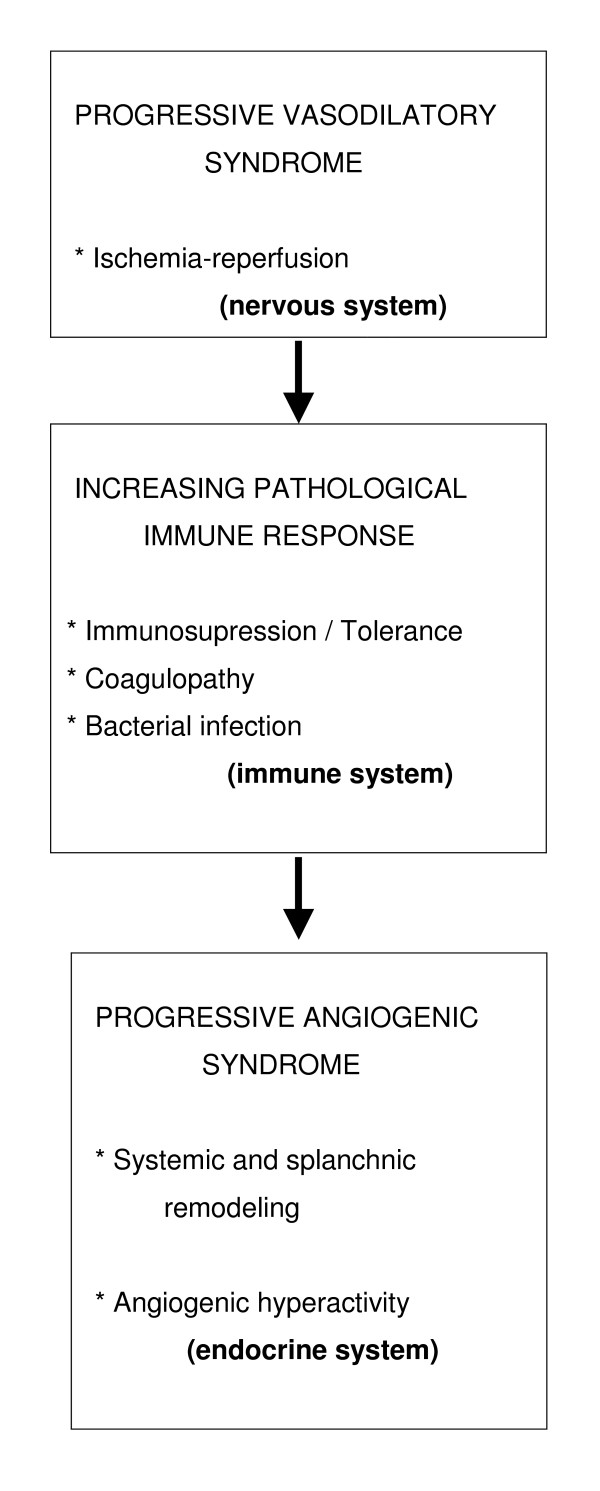
**Hypothetical evolution of the inflammatory systemic response when portal hypertension is associated with chronic liver disease**. In the chain expression of the three proposed functional systems (nervous, immune and endocrine) mast cells and their mediators could participate.

Therefore, the relationship between the liver anti-oxidative capacity and the severity of the systemic complications could be more important than the grade of splanchnic and systemic oxidative stress. Aside from the degree of oxidative stress, the reduction of the hepatic anti-oxidant capacity would aggravate the intensity of the inflammatory response [34]. In this hypothetical situation, the progressive evolution of the chronic liver disease, would be associated with a progressive reduction of its anti-oxidant capacity and as a consequence, it would favor the systemic inflammatory response. Since the proposed inflammatory response is based on the successive functional predominance of the nervous, immune and endocrine systems [36,38] it could be considered that the worsening of the systemic hyperdynamic circulation associated with an ischemia-revascularization (nervous) phenomenon would favor the progression of tissue infiltration by inflammatory cells and bacteria (immune). Therefore, the remodeling of the body would become more intense, favoring both splanchnic and systemic angiogenesis (endocrine) (Figure 5).

Mast cells can significantly influence multiple factors of chronic inflammatory responses, though diverse effects that can either promote or, perhaps more surprisingly, suppress aspects of these responses [138]. In the case of the chronic liver disease complicating the evolution of portal hypertension, mast cells could participate as regulators of the inflammatory response evolution. And so, during the evolution of this disease, harmful factors can position or "tune" mast cells within a broad spectrum of functional responsiveness [46] that would favor or restrict the progression of the inflammatory response.

**Figure 6:**
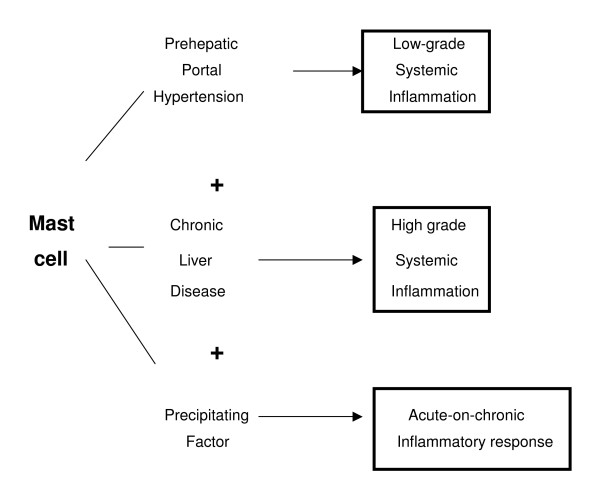
**Inflammatory phenotypes**. Possible types of the inflammatory response, where mast cells play the main role during the evolution of chronic liver disease.

Chronic liver disease could favor the expression in the body of a dedifferentiation process. Through this defensive mechanism, the functions with a higher energy requirement would be reduced [36,38]. In particular, the specialized epithelial cells have high energy costing functions. These epithelial cells have nutrition mediated by blood capillaries. The combination of elevated capillary support of oxygen with the oxidative metabolism (oxidative phosphorylation) allows epithelial cells to obtain an abundant energy supply, which is used to drive multiple specialized processes with limited heat generation (coupled reaction) [38]. In the evolution of the chronic liver disease, the systemic inflammatory response could represent the establishment of lesser energy "costing" functions, since using oxygen to get energy is not as effective (uncoupled reactions). In this hypothetical situation, the mast cells would collaborate in the expression of the inflammatory phenotype most appropriate according the needs of the body (Figure 6).

Therefore, the mast cells could have a defensive and favoring role in high-risk survival situations. The incidence of harmful influences during the evolution of chronic liver disease could involve the regression to the most primitive metabolic stages, and the adoption of an ischemic-reperfusion phenotype (nervous system). The body, under the effects of an ischemia-reperfusion phenomenon, reduces its metabolic needs (oxidative stress), favors its hydration and salinization (water and salt retention) and enhances nutrition by diffusion (interstitial and cellular edema). These supposed defense mechanisms are simple but also less costly, and facilitate temporary survival until a more favorable environment makes it possible to initiate more complex metabolic and nutritional functions. Thus, this would explain why both patients and animals with chronic liver disease and portal hypertension are predisposed to develop hepatorenal syndrome, ascites and pleural effusion when they suffer harmful influences [10,11,140]. In essence, they would suffer a process of reperfusion, a major complication in which mast cells could participate since they have the right mediators for inducing this acute-on-chronic pathology in patients and animals with portal hypertension associated with chronic liver diseases.

## Conclusion

In conclusion, mast cells could participate in the production of a low-grade systemic inflammatory response that induces portal hypertension. Mast cells also participate in the worsening of the inflammatory response when a chronic liver disease is associated. Finally, these fascinating cells collaborate in the decompensation of the systemic inflammatory reaction or acute-on-chronic response if a precipitating factor prevails.

## Abbreviations

AP-1: Activator Protein-1; CCl_4_: Carbon Tetrachloride; CO: Carbon monoxide; eNOS: endothelial Nitric Oxide Synthase; H_2_S: Hydrogen Sulfide; HIF-1: Hypoxia Inducible Factor-1; NO: nitric oxide; NF-κB: Nuclear Factor Kappa B; PARs: Protease-activated receptors; PGI_2_: Prostacyclin; PVL: partial portal vein ligation; ROS/RNS: Reactive Oxygen and Nitrogen Species; RMCP-II: Rat Mast Cell Protease-II; SDF-1: Stromal-derived Factor; TAA: Thioacetamide; TGF-β: Transforming Growth Factor-beta; TNF-α: Tumor Necrosis Factor alpha; t-PA: tissue type Plasminogen Activator; VEGF: Vascular Endothelial Growth Factor.

## Competing interests

The author(s) declare that they have no competing interests.

## Authors' contributions

The three authors conceived, discussed and wrote the manuscript.
